# Left cardiac sympathetic denervation in the Netherlands for the treatment of inherited arrhythmia syndromes

**DOI:** 10.1007/s12471-014-0523-2

**Published:** 2014-02-13

**Authors:** L. R. A. Olde Nordkamp, A. H. G. Driessen, A. Odero, N. A. Blom, D. R. Koolbergen, P. J. Schwartz, A. A. M. Wilde

**Affiliations:** 1 Heart Center, Department of Cardiology, Academic Medical Centre, PO Box 22700, 1100 DE Amsterdam, the Netherlands; 2 Heart Center, Department of Cardiothoracic Surgery, Academic Medical Centre, Amsterdam, the Netherlands; 3 Department of Lung, Blood and Heart, University of Pavia, Pavia, Italy; 4 Department of Paediatric Cardiology, Academic Medical Centre, Amsterdam, the Netherlands; 5 Center for Cardiac Arrhythmias of Genetic Origin, IRCCS Instituto Auxologico Italiano, Milan, Italy; 6 Department of Molecular Medicine, University of Pavia, Pavia, Italy; 7 Cardiovascular Genetics Laboratory, Hatter Institute for Cardiovascular Research in Africa, Department of Medicine, University of Cape Town, Cape Town, South Africa; 8 Department of Medicine, University of Stellenbosch, Stellenbosch, South Africa; 9 Department of Family and Community Medicine, College of Medicine, King Saud University, Riyadh, Saudi Arabia; 10 Princess Al-Jawhara Albrahim Centre of Excellence in Research of Hereditary Disorders, King Abdulaziz University, Jeddah, Saudi Arabia

**Keywords:** Left cardiac sympathetic denervation, Inherited arrhythmia syndromes, Ventricular arrhythmias

## Abstract

**Introduction:**

Treating therapy-resistant patients with inherited arrhythmia syndromes can be difficult and left cardiac sympathetic denervation (LCSD) might be a viable alternative treatment option. We provide an overview of the indications and outcomes of LCSD in patients with inherited arrhythmia syndromes in the only tertiary referral centre in the Netherlands where LCSD is conducted in these patients.

**Methods:**

This was a retrospective study, including all patients with inherited arrhythmia syndromes who underwent LCSD in our institution between 2005 and 2013. LCSD involved ablation of the lower part of the left stellate ganglion and the first four thoracic ganglia.

**Results:**

Seventeen patients, 12 long-QT syndrome (LQTS) patients (71 %) and 5 catecholaminergic polymorphic ventricular tachycardia (CPVT) patients (29 %), underwent LCSD. Most patients (94 %) were referred because of therapy-refractory cardiac events. In 87 % the annual cardiac event rate decreased. However, after 2 years the probability of complete cardiac event-free survival was 59 % in LQTS and 60 % in CPVT patients. Two patients (12 %) had major non-reversible LCSD-related complications: one patient suffered from a Harlequin face post-procedure and one severely affected LQT8 patient died the day after LCSD due to complications secondary to an arrhythmic storm during the procedure.

**Conclusion:**

LSCD for inherited arrhythmia syndromes, which is applied on a relatively small scale in the Netherlands, reduced the cardiac event rate in 87 % of the high-risk patients who had therapy-refractory cardiac events, while the rate of major complications was low. Therefore, LSCD seems a viable treatment for patients with inherited arrhythmia syndromes without other options for therapy.

## Introduction

Primary inherited arrhythmia syndromes, such as long-QT syndrome (LQTS) and catecholaminergic polymorphic ventricular tachycardia (CPVT), are genetic cardiac diseases without apparent structural heart disease that may cause cardiac syncope and sudden cardiac death, mainly in young individuals. These cardiac events are generally induced by physical or emotional stress triggers [1–3]. Hence, β-blockers are considered first-line therapy in symptomatic and asymptomatic patients in both conditions [4, 5]. In addition, an implantable cardiac defibrillator (ICD) is often used in patients who continue to have ventricular arrhythmias despite β-blocker therapy [5, 6]. However, ICDs do not *prevent* ventricular arrhythmias and can even trigger catecholamine release, subsequently resulting in arrhythmic storms and even death [7]. Also the cost of frequent shocks in terms of pain and fear is substantial [8] and young patients with ICDs are more likely to experience device complications, including inappropriate shocks and lead-related complications, over many years of use [9].

In 1971, Moss and McDonald [10] described left cardiac sympathetic denervation (LCSD), which prevents norepinephrine release in the heart, therefore raising the threshold for ventricular fibrillation without reducing the heart rate or impairing myocardial contractility [11]. In the last decade LCSD has received renewed attention as a viable alternative treatment for therapy-resistant LQTS and CPVT patients. A significant protective effect of LCSD was demonstrated in both symptomatic and asymptomatic LQTS and CPVT patients [12–16]. In the Netherlands, LCSD is applied on a small scale and we hereby provide an overview of the indications and outcomes of LCSD in patients with inherited arrhythmia syndromes in the only tertiary referral centre where LCSD is conducted.

## Methods

### Study design

Patients who received LCSD in our hospital between 1 November 2005 and 1 February 2013 for LQTS or CPVT were included in this study. LQTS was defined according to the diagnostic criteria described by Schwartz et al. [17] The QT interval was assessed from lead II/V5 and corrected for heart rate using Bazett’s formula. CPVT was diagnosed based on exercise-induced bidirectional or polymorphic ventricular tachycardia (VT) in the absence of structural cardiac disease and a putative pathogenic mutation in a CPVT-causing gene [2].

All data available prior to and after the LCSD were retrospectively extracted from medical records. These included demographic data, clinical data (initial presentation, age at onset, family history of sudden cardiac death (SCD) <60 years), genotype, ECG data, presence of ICD and medical therapy, incidence of cardiac events and indication for surgery. Cardiac events were defined as cardiac syncope, aborted cardiac arrest (ACA), appropriate ICD shocks or malignant non-sustained ventricular tachycardias (NSVT). LSCD-related data included procedure time, length of hospital stay and postoperative complications. The surgery was considered secondary prevention if the patient had a history of cardiac events. The institutional review board of our institution waived the requirement for informed consent.

### Surgical procedure

In the first group of patients (*n* = 6), the surgical procedure for LCSD started with an incision at the base of the neck (supraclavicular approach [18], performed by A.O., A.D. and D.K.), which is an extrapleural approach without opening the chest. The other patients underwent a video-assisted thoracoscopic LCSD [19], except for one 2-month-old baby who underwent denervation by thoracotomy (performed by A.D. and D.K.). In both the supraclavicular and video-assisted thoracoscopic approach, the lower part of the stellate ganglion (preferably dissection along the anatomical fusion between the upper and lower pole) was removed together with the second and third thoracic ganglia; the fourth ganglion was cauterised. Additional visible nerve structures from the sympathetic ganglia towards the heart were also cauterised. It provides adequate cardiac denervation with no or minimal Horner’s syndrome, because the upper half of the stellate ganglion is preserved.

### Statistics

All data were analysed with SPSS (19.0 SPSS Inc, Chicago, IL). Categorical data are displayed as percentage and compared between groups using a χ^2^ test. Normally distributed continuous data, tested for normality with the Shapiro-Wilk test, were described as mean (standard deviation [SD]). Continuous data not normally distributed were expressed as median (interquartile range [IQR]) and compared between groups using the Mann-Whitney *U*-test. Annual event rates were calculated as the number of events per year of observation. Postoperative event-free survival was described by Kaplan-Meier cumulative estimates. Confidence intervals for the median number of episodes and the median annual event rates were based on the method proposed by Bonett and Price [20]. The patient who died a few days after LCSD was removed from the denominator in the cardiac event analysis after LCSD, since follow-up was not possible. *P*-values <0.05 were considered statistically significant.

## Results

### Patient characteristics

From November 2005 to January 2013, 17 patients (12 LQTS patients (71 %) and 5 CPVT patients (29 %)) had a LCSD performed at our institution. Clinical characteristics of these patients are summarised in Table 1. The mean age of the entire study population at the time of LCSD was 19 ± 14 years and 59 % were female. All patients were on β-blocking therapy and three of the five CPVT patients had additionally taken flecainide prior to LCSD. Eight patients (47 %), of whom seven were diagnosed with LQTS, had already ICD implanted by the time of the surgery and one LQTS patient received an ICD 1 month after LCSD.

**Table 1:** Patient characteristics

	Total	LQTS	CPVT
Total number of patients	17	12	5
Female	10 (59 %)	7 (58 %)	3 (60 %)
Family history of sudden cardiac death <60 years	5 (29 %)	2 (17 %)	3 (60 %)
Genotype			
▪ KCNQ1 mutation (LQT1)	2 (12 %)	2 (17 %)	0
▪ KCNH2 mutation (LQT2)	6 (35 %)	6 (50 %)	0
▪ SCN5A mutation (LQT3)	2 (12 %)	2 (17 %)	0
▪ CACNA1C mutation (LQT8)	1 (5.9 %)	1 (8.3 %)	0
▪ LQTS of unknown type	1 (5.9 %)	1 (8.3 %)	0
▪ RyR2 mutation	5 (29 %)	0	5 (100 %)
Presentation			
▪ Aborted cardiac arrest / VT in history	6 (35 %)	4 (33 %)	2 (40 %)
▪ Cardiac syncope	6 (35 %)	4 (33 %)	2 (40 %)
▪ Family history	4 (24 %)	3 (25 %)	1 (20 %)
▪ Medical evaluation, other	1 (5.9 %)	1 (8.3 %)	0
Age at first cardiac event (median)	11 (IQR 0–18)	12 (IQR 0–17)	10 (IQR 2–19)
Age at LCSD (mean)	19 ± 14	20 ± 16	17 ± 4
Indication for LCSD			
▪ Primary prevention	1 (5.9 %)	1 (8.3 %)	0
▪ Secondary prevention	16 (94 %)	11 (92 %)	5 (100 %)
Prior therapies			
▪ β-blocker	17 (100 %)	12 (100 %)	5 (100 %)
▪ Mexiletine	2 (12 %)	2 (17 %)	0
▪ Flecainide	4 (24 %)	1 (8.3 %)	3 (60 %)
ICD	9 (53 %)	8 (67 %)	1 (20 %)
QTc			
▪ Pre-LCSD (median)	460 (IQR 395–496)	477 (IQR 413–526)	390 (IQR 381–442)
▪ Post-LCSD (median)	450 (IQR 413–504)	459 (IQR 440–538)	402 (IQR 348–464)

*CPVT* catecholaminergic polymorphic ventricular tachycardia, *ICD* implantable cardiac defibrillator, *IQR* interquartile range, *LCSD* left cardiac sympathetic denervation, *LQTS* long-QT syndrome

**Fig. 1:**
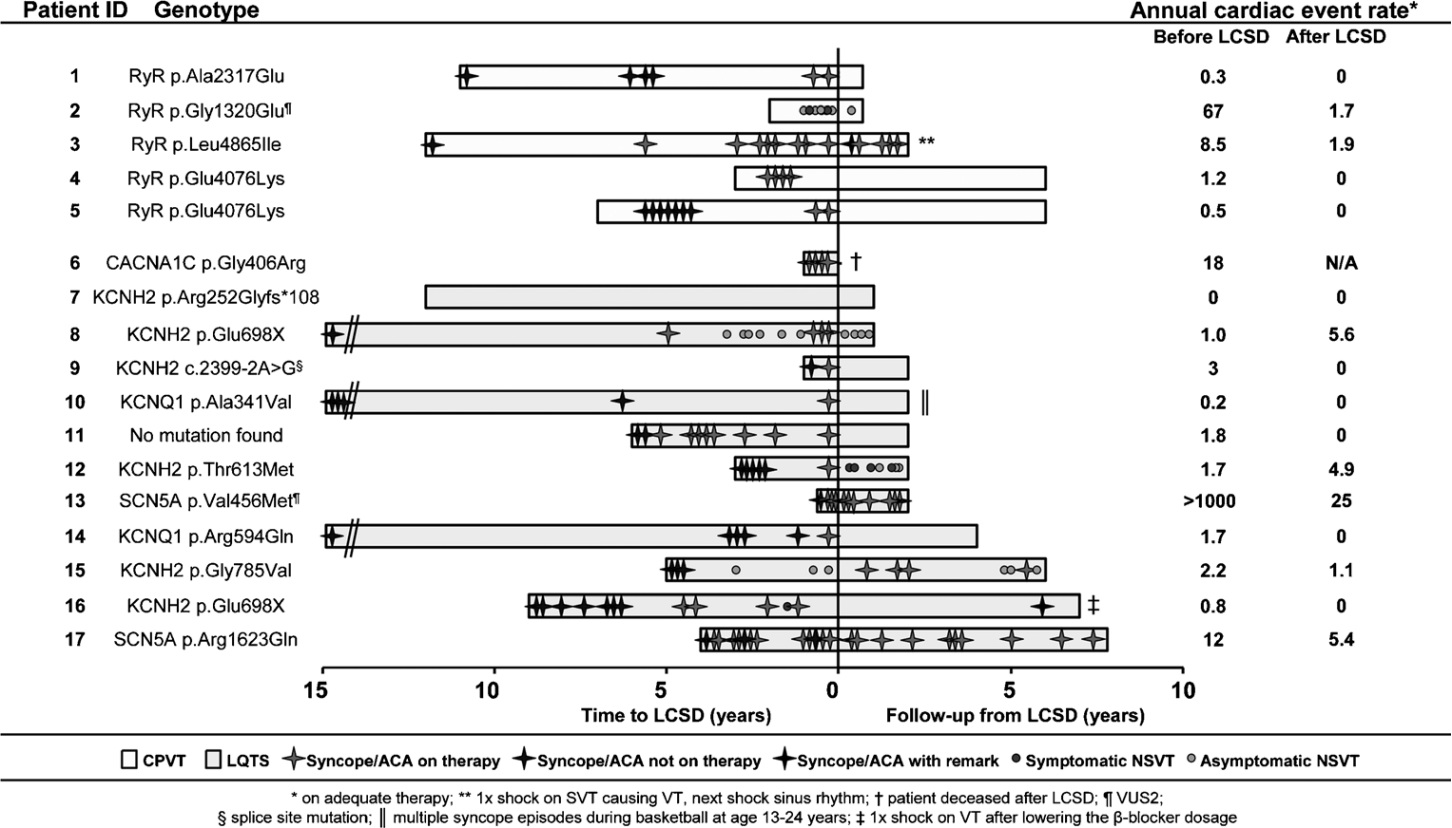
Comparison of cardiac events before and after LSCD

All CPVT patients had a mutation in the RyR2 gene, which were classified as putatively pathogenic in four patients. One patient (#2 in Fig. 1) had an unclassified variant in the RyR2 gene. Of the patients with LQTS, six (50 %) had a mutation in the KCNH2 gene, and two patients (17 %) had LQTS type 1 based on a mutation in the KCNQ1 gene. One LQTS patient (#11) was tested for all known LQTS genes, but no mutation was found.

### Indications

Sixteen patients (94 %) underwent LCSD after experiencing cardiac events under adequate medical therapy (9 [53 %] VT/VF, 5 [29 %] syncope and 2 [12 %] malignant NSVT), while one patient (5.9 %) underwent LCSD for primary prevention because of β-blocker intolerance. Figure 1 demonstrates the event rate timing prior to LCSD.

### LCSD procedure

Six (35 %) patients underwent LCSD using the supraclavicular approach and in 10 patients (58 %) LCSD was performed via a video-assisted thoracoscopic approach. One 2-month-old baby underwent LCSD via thoracotomy because a video-assisted thoracoscopic approach was not deemed feasible. The median skin-to-skin time was 46 (IQR 39-61) minutes, with a median time of 40 (IQR 34–78) minutes for the supraclavicular approach and 47 (IQR 43–67) minutes for the thoracoscopic approach (*p* = 0.33). The median number of hospitalisation days after the procedure was 3 (IQR 2–7) days. There was no difference in number of hospitalisation days between the two surgical approaches (supraclavicular approach 3 (IQR 3–23) days and thoracoscopic approach 2 (IQR 2–4) days; *p* = 0.13).

Major non-reversible complications of the LCSD were reported in 2/17 (12 %) of the patients. One patient (5.9 %) suffered from a postoperative Harlequin face. One severely affected LQT8 patient (5.9 %; patient #6 in Fig. 1) with multiple arrhythmic storms before LCSD died after the procedure, due to multi-organ failure secondary to bradycardias followed by arrhythmic storms in combination with severe electrolytic disturbances and hypoglycaemia during and after the procedure. Minor complications of the LCSD, which spontaneously resolved, were reported in four (24 %) additional patients: three of these patients (18 %), all treated using the video-assisted thoracoscopic approach, were diagnosed with a post-procedural pneumothorax and one patient (5.9 %) had a transient postoperative Horner’s syndrome.

### Cardiac events after LCSD

During a median follow-up of 34 (IQR 16–77) months, 7 out of 15 (47 %) *symptomatic* patients did not experience any occurrence of cardiac events after LCSD (Fig. 1). Cardiac events continued in 8/15 (53 %) symptomatic patients (Fig. 1 and Table 2). In one of these patients with recurrences, an ICD shock occurred after a decrease in β-blocker dosage and in two patients only asymptomatic NSVT persisted, while they suffered from life-threatening cardiac syncope and ICD shocks before LCSD. Additionally, one patient (#17), who was diagnosed with LQT3 which overlapped with Brugada syndrome, had recurrences of life-threatening cardiac events after LCSD, but these events were mainly fever-related and thus probably related to Brugada syndrome. Life-threatening cardiac events persisted at a high(er) annual event rate after LCSD in only one patient. This LQT2 patient (#12) experienced multiple appropriate ICD shocks after LCSD during the postpartum period. When analysing the video-assisted thoracic surgery footage in retrospect, the removal of the lower part of the stellate ganglion was insufficiently cranial. In general, cardiac events on adequate medical therapy recurred in 0 out of 2 LQT1 patients, three out of six LQT2 patients, two out of two LQT3 patients (*n* = 2/2), and two out of five of the CPVT patients.

**Table 2:** Patient characteristics of patients with cardiac events after LCSD while on adequate medical therapy

Patient ID	Sex	Disease	Age of onset	Age LCSD	Number of CE before LCSD^a^	Number of CE after LCSD	Event circumstance	QTc before/after LCSD
2	Female	CPVT	21	21	>100^b^	1^b^	Exercise	383/423 ms
3	Male	CPVT	3	15	>100	4	Exercise	390/402 ms
8	Female	LQT2	16	35	>10	7^b^	During rest, waking up or in the evening	460/441 ms
12	Female	LQT2	16	19	3	>10^b^	Sudden emotion/noise	533/439 ms
13	Male	LQT3	Birth	0	>100	>50	Neonatal, during rest or suddenly	654/642 ms
15	Female	LQT2	20	25	9	7	Sudden emotion/noise and postpartum period	506/503 ms
16	Female	LQT2	12	21	5	1^c^	During rest, waking up or suddenly	485/505 ms
17	Male	LQT3 and BrS	Birth	4	>50	>10	Neonatal, playing and during fever	535/352 ms

*BrS* Brugada syndrome, *CPVT* catecholaminergic polymorphic ventricular tachycardia, *LCSD* left cardiac sympathetic denervation, *LQTS* long-QT syndrome

^a^Under adequate medication

^b^Only short non-sustained ventricular tachycardia (<10 complexes)

^c^Cardiac event (CE) after LCSD was after decrease in β-blocker dosage

In total, in 13/15 symptomatic patients (87 %) the annual cardiac event rate decreased importantly after LCSD. The median number of cardiac events decreased from five (95 % confidence interval [CI]: 0–22) to 0 (95 % CI: 0–3.5). The median annual event rate decreased from 1.7 (95 % CI: 0–5.6) to 0 (95 % CI: 0–1.0).

**Fig. 2:**
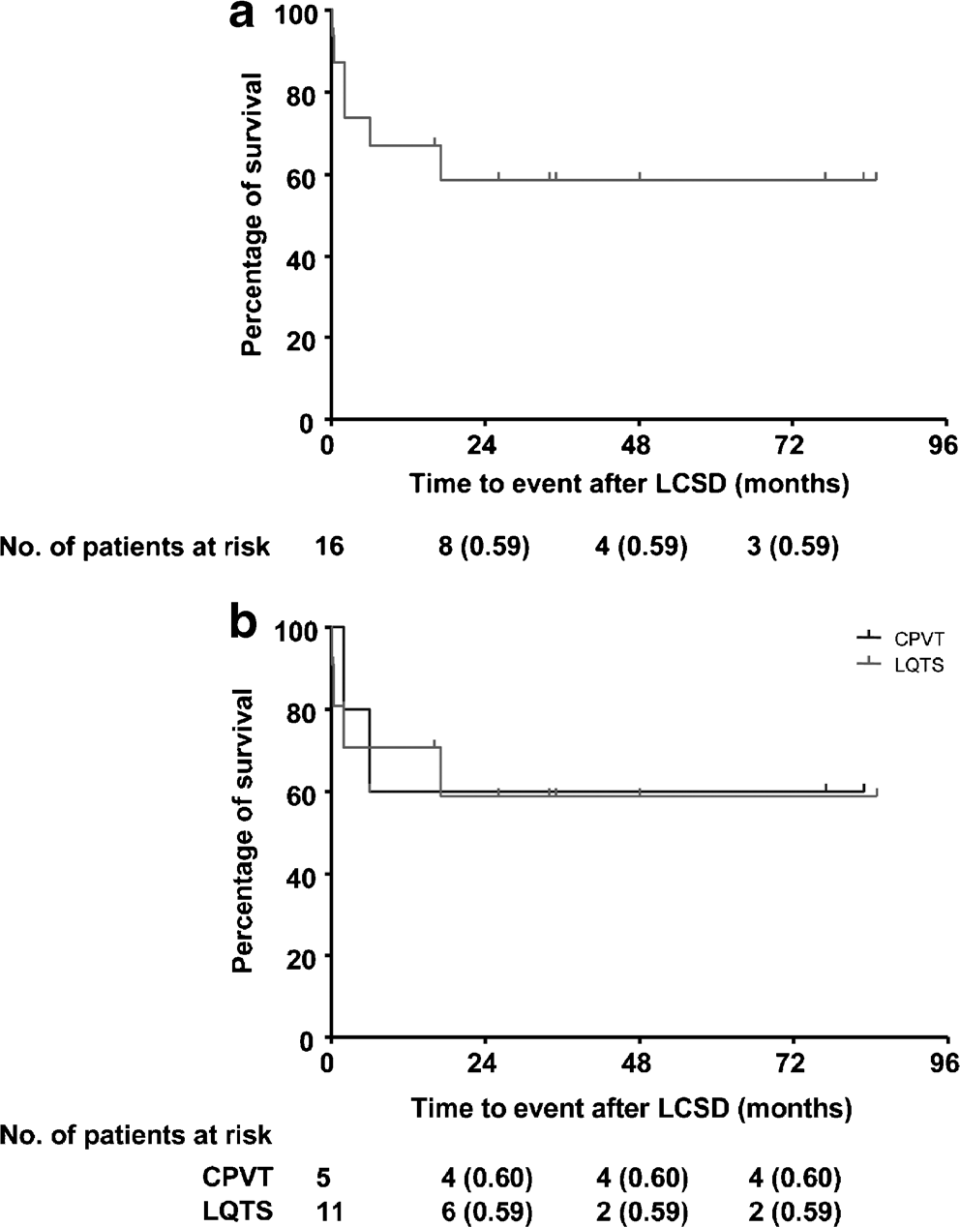
**a** Kaplan-Meier curves of event-free survival after LCSD: **a** all patients and **b** by disease

Kaplan-Meier estimates of postoperative cardiac event-free survival in all patients and by disease are shown in Fig. 2. Patients with LQTS had a 71 % and 59 % probability of event-free survival at 1 and 2 years, respectively, while these rates were 60 % after both 1 and 2 years in CPVT patients.

## Discussion

Today, LCSD is increasingly recognised as a viable treatment for therapy-resistant LQTS and CPVT patients [13–15]. Here we report the experience in the only tertiary referral centre in the Netherlands for this treatment in 17 patients with inherited arrhythmia syndromes. Most patients (94 %) were referred because of therapy-refractory cardiac events. In 87 % of the symptomatic patients, the annual cardiac event rate decreased. However, after 2 years the probability of cardiac event-free survival was 59 % in LQTS and 60 % in CPVT patients. There were four patients (24 %) with minor reversible complications who did not require any intervention, which is a similar number compared with other centres [14, 15, 21], and one patient (5.9 %) with a non-reversible post-procedural Harlequin face. Also, one LQT8 patient (5.9 %) died after the LCSD procedure due to complications secondary to bradycardias followed by an arrhythmic storm in combination with electrolytic disturbances and hypoglycaemia during the procedure. An identical case was reported by Schwartz et al. [12] in 1991, where a 3-year-old female patient died early postoperatively with suspected post-anaesthesia hypoxic distress (hypoglycaemia, respiratory failure, complete heart block and asystole). This patient was later identified as a LQT8 patient, and extreme caution should be taken in this type of patients.

### Success of LCSD

Our results are in agreement with previously published experience. Indeed, multiple studies have reported a significant decrease of cardiac events in 90 % of the symptomatic patients with inherited arrhythmia syndromes after LCSD, although approximately 40 % remained symptomatic [13–15, 19, 21–23]. The antiarrhythmic and antifibrillatory effects of LCSD are attributed to the reduced release of norepinephrine at the level of the ventricles [11]. This sympathetic blockade prolongs ventricular refractoriness and increases the ventricular fibrillation threshold, resulting in the prevention or suppression of triggered activity [11]. The denervation has a highly specific antiarrhythmic effect, without reducing the heart rate (which might be especially important for LQT3 patients) and without impairing myocardial contractility [11]. Additionally, because the denervation does not completely eliminate catecholamine input to the heart, surgery does not lead to hypersensitivity [11]. And lastly, re-innervation is not expected because the denervation is pre-ganglionic, which makes it likely that the effects are durable [18]. However, especially in highly symptomatic patients with inherited arrhythmia syndromes, LCSD has so far been advocated merely as an adjuvant therapy besides β-blockers (in LQTS and CPVT) or flecainide (in CPVT), as also demonstrated by the breakthrough cardiac event of patient #16 after lowering the β-blocker dosage. Indeed, ICDs should still be considered in patients at high risk for SCD.

Recently, percutaneous renal sympathetic denervation has emerged as a therapeutic option for patients with hyperactivity of the sympathetic system such as therapy-resistant hypertension [24]. Additionally, in animal models renal denervation suppressed ventricular arrhythmias [25]. These findings support the hypothesis that renal denervation might also be useful in reducing sympathetic activity in highly symptomatic patients with inherited arrhythmia syndromes and thereby reducing cardiac event rate, although this needs to be studied in the future.

### Response to LCSD among the different genotypes

Although there was a significant decrease in cardiac events in most patients, half of the patients remained symptomatic. Post-LCSD cardiac events recurred in 3/6 of the LQT2 patients, 2/2 of the LQT3 patients, and 2/5 of the CPVT patients, while none of the LQT1 patients had events. The number of patients is too small to draw any meaningful conclusions, but the various results among the different genotypes are in agreement with the underlying pathophysiological mechanism. During gradual progressive sympathetic activation (such as exercise), the reduced, catecholamine-sensitive, slow delayed rectifier potassium current (I_Ks_) in LQT1 patients prevents the necessary QT adaptation, causing a risk for triggered activity and ventricular arrhythmias [26, 27]. Therefore, removing the sympathetic activation of the heart with LCSD appeared to be very successful in LQT1 patients [15].

On the other hand, cardiac events in LQT2 patients are often triggered by a sudden heart rate acceleration, such as during a loud noise or surprising emotion [28]. In LQT2 patients with a reduced rapid delayed rectifier potassium current (I_Kr_), possible extrasystoles triggered by these triggers result in a markedly pause-dependent prolongation of the action potential duration of the following heart beat and attendant risk of early afterdepolarisations [27, 29]. Sudden heart rate increment is caused by vagal withdrawal rather than sympathetic activation [30] and this might explain that removal of the sympathetic activation by LCSD is not so successful in all LQT2 patients. Also the occurrence of extrasystoles might not be prevented.

In both LQT3 patients, the number of cardiac events decreased, but the effect of LCSD was not complete. Also, failure of complete LCSD success in CPVT may be attributed to the fact that only the local, cardiac, release of catecholamines is blocked. Systemic release of catecholamines is still pertinent and may be sufficient to elicit triggered arrhythmias during physical exercise, despite β-blockade and flecainide therapy. Furthermore, one CPVT patient (#2) with recurrent events carries an unclassified variant in the RyR2 gene, which might indicate that the diagnosis CPVT might not be correct.

Although the success of LCSD was not complete among the different genotypes, the number of events decreased in almost all patients (87 %), thus significantly improving the quality of life of the patients and their families. Therefore, LCSD continues to be worthwhile in highly symptomatic patients with primary arrhythmias.

### Limitations

This is retrospective study with a small number of patients, secondary to the rare prevalence of therapy-resistant LQTS and CPVT patients. Due to the retrospective nature, a quantitative marker of arrhythmia burden has been difficult to express in statistically comparable data due to the large variability in presenting symptoms, resulting in a wide definition of cardiac events. Also, in patients without an ICD, the lack of continuous heart rate monitoring could have resulted in an underestimation of cardiac events, especially NSVT. However, cardiac event monitoring before and after LCSD was identical in all patients except for one LQT patient who received an ICD 1 month after LCSD.

## Conclusion

LSCD for inherited arrhythmia syndromes, although applied on a small scale in the Netherlands, reduced the cardiac event rate in 87 % of the high-risk patients who had therapy refractory cardiac events. Although one severely affected LQT8 patient died secondary to surgery-related issues, LSCD seems a viable treatment for patients with inherited arrhythmia syndromes without other options for therapy.
